# Improvements throughout the Three Waves of COVID-19 Pandemic: Results from 4 Million Inhabitants of North-West Italy

**DOI:** 10.3390/jcm11154304

**Published:** 2022-07-25

**Authors:** Valeria Caramello, Alberto Catalano, Alessandra Macciotta, Lucia Dansero, Carlotta Sacerdote, Giuseppe Costa, Franco Aprà, Aldo Tua, Adriana Boccuzzi, Fulvio Ricceri

**Affiliations:** 1 High Dependency Unit, Emergency Department, San Luigi Gonzaga University Hospital, 10043 Orbassano, TO, Italy; v.caramello@sanluigi.piemonte.it (V.C.); a.boccuzzi@sanluigi.piemonte.it (A.B.); 2 Department of Clinical and Biological Sciences, University of Turin, 10043 Orbassano, TO, Italy; alessandra.macciotta@unito.it (A.M.); lucia.dansero@unito.it (L.D.); giuseppe.costa@unito.it (G.C.); fulvio.ricceri@unito.it (F.R.); 3 Unit of Cancer Epidemiology, Città Della Salute e Della Scienza University Hospital, 10126 Turin, TO, Italy; carlotta.sacerdote@cpo.it; 4 Unit of Epidemiology, Regional Health Service ASL TO3, 10095 Grugliasco, TO, Italy; 5 High Dependency Unit, Emergency Department, San Giovanni Bosco Hospital, 10154 Turin, TO, Italy; franco.apra@aslcittaditorino.it; 6 Emergency Department, Azienda Sanitaria Locale Biella, 13875 Ponderano, BI, Italy; aldo.tua@aslbi.piemonte.it

**Keywords:** SARS-CoV-2, mortality, intensive care units, patients, comorbidity, epidemiology

## Abstract

At the very beginning of the European spread of SARS-CoV-2, Piedmont was one of the most affected regions in Italy, with a strong impact on healthcare organizations. In this study, we evaluated the characteristics and outcomes of the COVID-19 patients in an entire region during the first three pandemic waves, identifying similarities and differences in the SARS-CoV-2 epidemic’s timeline. We collected the health-administrative data of all the Piedmont COVID-19 patients infected during the first three pandemic waves (1 March 2020–15 April 2020; 15 October 2020–15 December 2020; 1 March 2021–15 April 2021, respectively). We compared differences among the waves in subjects positive for SARS-CoV-2 and in patients admitted to ICU. Overall, 18.621 subjects tested positive during the first wave (405 patients/day), 144.350 (2366.4 patients/day) in the second, and 81.823 (1778.8 patients/day) in the third. In the second and third waves, we observed a reduction in median age, comorbidity burden, mortality in outpatients, inpatients, and patients admitted to ICU, in intubation, invasive ventilation and tracheostomy, and a parallel increase in the use of CPAP. Our study confirmed a trend towards younger and healthier patients over time but also showed an independent effect of the period on mortality and ICU admission. The appearance of new viral variants, the starting of vaccination, and organizational improvements in tracking, outpatients and inpatients management could have influenced these trends.

## 1. Introduction

**Figure 1:**
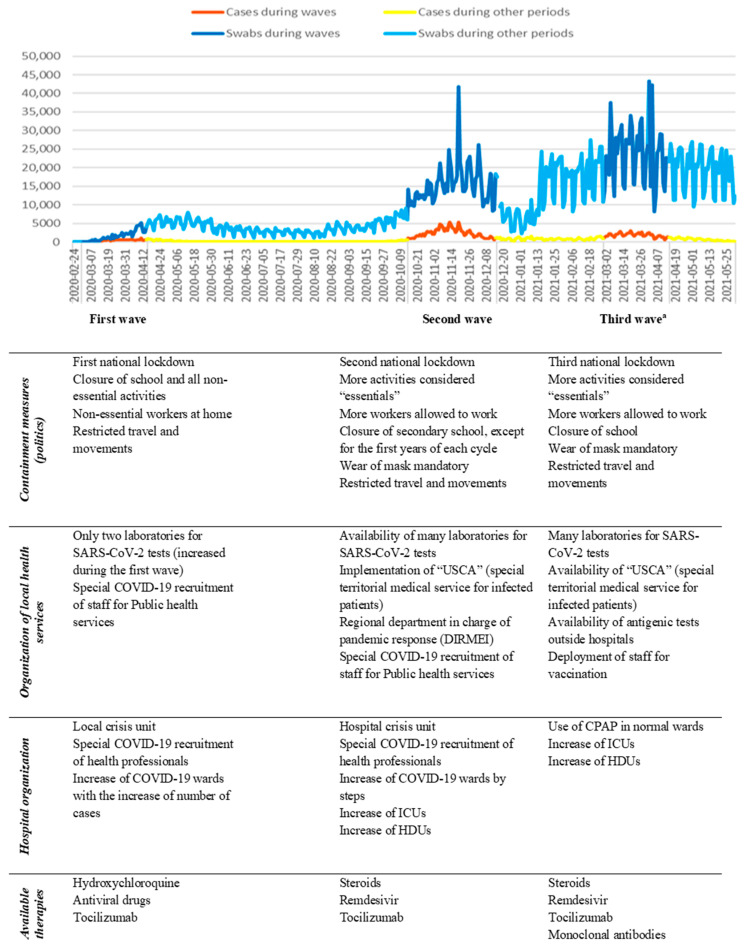
Number of swabs performed, number of new SARS-CoV-2 positive cases registered in Piedmont during the COVID-19 pandemic, and main interventions issued by the Italian Government and the health care system. (Data source: GitHub—pcm-dpc/COVID-19: COVID-19 Italia—Monitoraggio situazione). ^a^ Spread of Delta variant.

Since the Severe Acute Respiratory Syndrome Coronavirus 2 (SARS-CoV-2) was declared a pandemic in March 2020 [1], Italy was the first country outside China and the first European country to be strongly impacted by the COVID-19 pandemic, which resulted in a significant health burden. Since the first case was reported in Lombardy on the 20th of February, the infection spread very quickly in the Northern regions and the densely populated area around the Po River by the end of March, with an observed increase in 29.5% from the expected mortality. In Italy, Piedmont (Figure S1) was the second affected region during the first wave and suffered from hospital overload, shortage of healthcare resources and professionals, as well as a massive death toll [1]. A structural reorganization and improvements in resource allocation were needed to cope with the emergency, both at the hospital level and in primary care, managed by the regional Crisis Unit. The emergency lasted many months, with three following waves in the first 16 months. These waves (1 March 2020–15 April 2020; 15 October 2020–15 December 2020; 1 March 2021–15 April 2021) were defined by a rising number of cases (405 cases/day; 2366.4 cases/day; 1778.8 cases/day, respectively) [1] with a corresponding increase in hospitalizations and deaths related to Coronavirus disease (COVID-19) and by the parallel restriction policies issued by the government [2] (Figure 1 and Figure S2). A summary of the main interventions is presented in Figure 1. As time passed, a better knowledge of COVID-19 physiopathology, clinical staging, and therapeutic possibilities was achieved, followed by an attenuation in the excess mortality [1,3,4] European Countries and, especially, Italy recorded an extremely high fatality rate (with a case fatality ratio up to 11%) [5,6] compared to China (Hubei region case fatality ratio of 4.7%) [7], which was the country where the pandemic started [3,4,8]. This is likely due to an underestimation of infection rates, the overload of ICUs, and the older age of infected patients during the first wave [4,8,9,10]. Many Italian authors observed a reduction in mortality, hospitalization, and ICU admission in the following second and third waves [9,10]. Others do not agree, especially when evaluating regions that were relatively spared or lightly affected during the first waves [8]. 

To the best of our knowledge, despite the worldwide spread of the disease, only a few studies have thoroughly compared the disease severity and the demographic and clinical characteristics of infected patients in subsequent waves [11,12,13]: these studies are monocentric [11,14,15] or on a regional [9,10,15,16,17] or national level [12,13,18] or concern specific populations [19]. 

Currently, the role of demographic and lifestyle factors and comorbid conditions on the risk of progression to severe COVID-19 is well defined; however, it is still controversial how different patient characteristics and the improvements in treatment and health care organization could contribute to morbidity and mortality. Moreover, public health institutions worldwide imposed different lockdown restrictions, tracing and control measures aiming, on one side, to reduce the number of cases and to protect the health system from overload or, on the other side, to achieve herd immunity. The systematic interpretation at a global level is challenging because of the enormous heterogeneity in sampling and methodology: many reports are based on mathematical modeling only [20], others are derived from different healthcare systems or are limited to a single medical center [9,10,14,15,19].

### Study Design and Aims

The aim of this large population-based region-wide study based on health administrative databases is to evaluate the characteristics and outcomes of the COVID-19 patients of the Piedmont Region during the three pandemic waves in an attempt to identify similarities and differences in the SARS-CoV-2 epidemic’s timeline during the first year of the Pandemic. 

Secondarily, in the subgroup of COVID-19 inpatients, we aimed to evaluate the differences in the need for respiratory support, for Intensive Care Unit (ICU), and the hospital length of stay to assess the severity of cases and the burden on Piedmont Hospitals resources. 

## 2. Materials and Methods

### 2.1. Study Population

**Figure 2:**
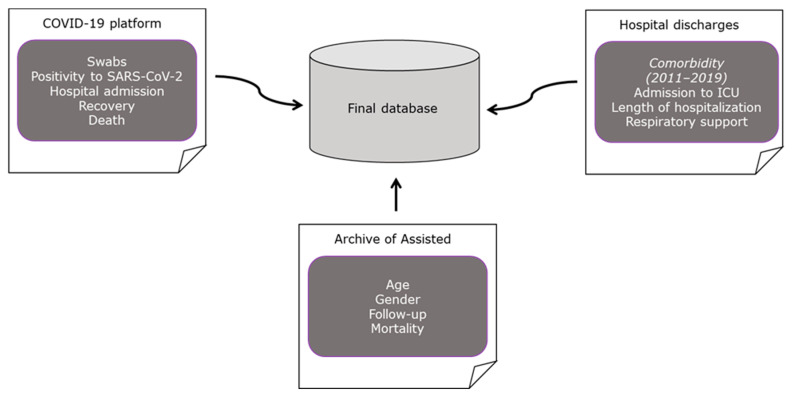
Flow chart on data collection process by record linkage of three different regional-health administrative archives.

To assess the characteristics of patients affected by COVID-19 for the first time during the three COVID-19 pandemic waves, data were obtained through record-linkage of regional health administrative data, as explained in Figure 2. 

The sources included were:

The regional platform “COVID-19”, in which data on subjects who had contact with the Piedmont health system related to SARS-CoV-2 (tested, quarantined, infected, sick, hospital admitted, dead or recovered) are collected;

The regional archive of Hospital Discharge Forms, which contains information on each patient from Piedmont discharged from public and private hospitalization institutions;

The Regional Unitary Archive of Assisted, which provides information on all those who applied for a general practitioner in the region and information on deaths.

The three COVID-19 pandemic waves were defined based on the number of cases (Figure 1) and on the restrictions implemented by the Italian Government, as follows: 

First wave: 1 March 2020–15 April 2020;

Second wave: 15 October 2020–15 December 2020; 

Third wave: 1 March 2021–15 April 2021.

### 2.2. Variables

Using data from the regional Archive of Hospital Discharge Forms related to a five-year period of 2015–2019, patients with previous neoplasia, diabetes, dementia, immunodeficiency, cardiomyopathy, heart failure and cardiovascular, coronary artery, cerebrovascular, haematologic, kidney, and chronic obstructive pulmonary diseases (COPD) have been identified. Moreover, for each subject, the value of the Charlson Comorbidity Index (CCI) [21] was calculated, and, only for hospitalized subjects, other information, such as the duration of hospitalization due to SARS-CoV-2 infection and the intervention procedures implemented were retrieved (oxygen, continuous positive airway pressure (CPAP), non-invasive ventilation (NIV), intubation, invasive ventilation, and tracheotomy). 

The outcome variables were defined as: hospitalization, hospitalization in ICU (both data were retrieved from the archives of hospital discharge forms), and mortality (retrieved by the combination of all the sources available).

### 2.3. Statistical Analysis

The data were described using the median, mean, interquartile range, and standard deviation or standardized frequencies, expressed as the average number of cases per day (due to the difference in length of the different waves) and percentages for quantitative and qualitative data, respectively. Moreover, for each clinical and sociodemographic variable, we tested possible significant differences between the three waves by using the Chi-square or Fisher test and ANOVA or Kruskal–Wallis test, both by considering all infected individuals and only the subjects admitted to the hospital for COVID-19, admitted to an intensive care unit (ICU) and who died within 30 days from the first positive swab.

To control for possible confounding factors, we evaluated the impact of the waves on the severity of COVID-19 using a logistic regression model for the combined outcome of ICU admission or mortality adjusted by age, gender, and comorbidity index. 

Analyses were performed using SAS (V9.4), SAS Institute Inc., Cary, NC, USA and R (V4.1.2), R foundation for statistical computing, Vienna, Austria.

## 3. Results

Among a population of about 4.3 million inhabitants, 357.436 tested positive for SARS-CoV-2 during the observed pandemic period (from 22 February 2020 to 31 May 2021). In the first wave, 18.621 subjects tested positive, which corresponds to a daily number of cases of about 405. During the second wave, the number of positive subjects increased to 144.350, corresponding to a daily number of 2366.4 cases. In the third wave, the number of positive decreased to 81.823, corresponding to a daily number of cases of 1778.8.

**Figure 3:**
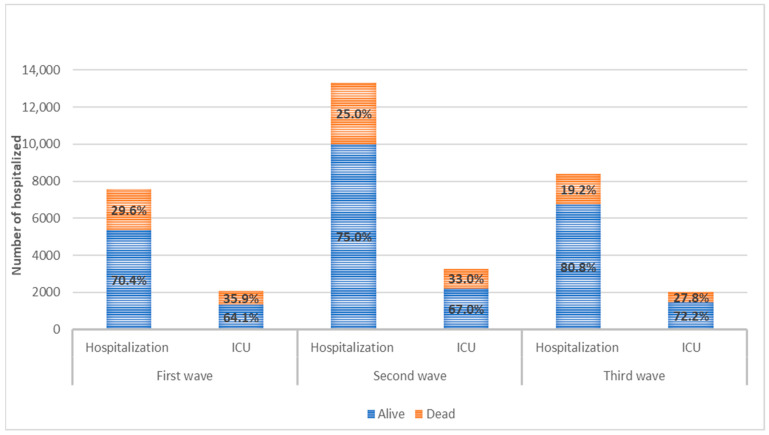
Mortality within 30 days from the first positive swab in hospitalized patients and in the subgroup of patients admitted to intensive care unit (ICU) in the three waves.

The descriptive data about the positive patients are presented in Table 1 and in Figure 3. In the first wave, the positive patients were older (I: 48.4% more than 65 years old; II: 24.1%; 21.5%, respectively), with more comorbidities (I: 41.0% with at least one comorbidity; II: 27.1%; III: 24.5%), and with more severe disease compared to the two following waves (higher percentage of hospitalizations, ICU admissions, and deaths). As expected, the daily number of cases of all categories considerably increased in the second waves and slightly decreased in the third waves; this is likely due to patients with no or mild symptoms, who were probably under-tested during the first wave. Indeed, outpatients in the second and third waves were, respectively, about 9 times and 6.5 times higher than in the first wave, and the increase in hospitalized patients was notably lower, being 1.3 and 1.1 times for the second and third waves. Interestingly, in the third wave, it was possible to observe a substantial decrease both in number and in the percentage of positive older subjects (>85 years old) (I: 15.8%; II: 6.3%; III: 2.9%), subjects with dementia (I: 3.8%; II: 1.1%; III: 0.3%), and dead patients (I: 17.9%; II: 3.9%; III: 2.6%), even if we compare the data with the first wave.

Patients who needed hospitalization (Table 2 and Table S1) in all the three waves were significantly older (I: patients admitted 65 + 64.4% vs. patients not admitted 65 + 37.4%; II: 70.0% vs. 19.4%; III: 64.1% vs. 16.6%)., of male gender (I: admitted 59.9% vs. not admitted 35.2%; II: 59.1% vs. 44.8%; III: 58.0% vs. 48.4%), and with more comorbidities (I: admitted with at least one comorbidity 51.6% vs. not admitted with at least one comorbidity 33.8%; II: 53.4% vs. 24.4%; III: 47.3% vs. 21.9%), but fewer differences could have been identified in hospitalized patients among the waves.

Differences among the three waves were less evident, both in absolute numbers and in percentages, considering patients hospitalized in ICU (Table 3 and Table S2). However, it is interesting to notice that in the third wave, a reduction in deaths was recorded both in the ICU and in the other wards.

Table 4 presents data regarding the therapeutic interventions received by patients once they were hospitalized, both in ICU and not in ICU. As expected, the length of hospitalization was longer in patients who were admitted to ICU, even if it seems that in the second wave, the length of hospitalization was shorter for those patients. About two out of three patients needed oxygen support during their hospitalization, provided both in ICU and in other wards. The need for CPAP significantly increased during the three waves (21.8%, in the first wave, 22.8% in the second wave, and 32.7% in the third wave), also because of a large increase in patients admitted out of the ICU. In the second and third waves, patients in ICUs underwent less intubation, invasive ventilation, and tracheostomy.

**Figure 4:**
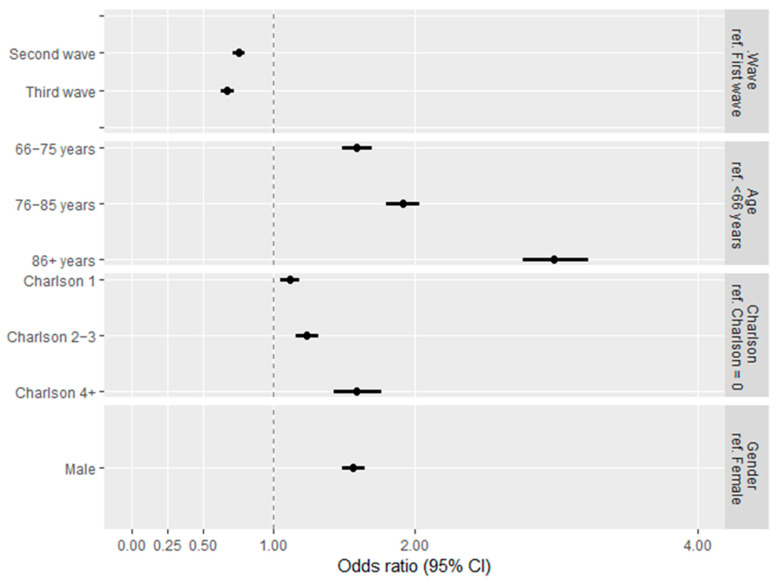
Odds ratio (OR) and 95% confidence interval (CI) estimates showing the effect of being infected in different waves on the combined outcome mortality and ICU admission, adjusting by age, gender, and comorbidity burden.

As expected, the patients who died were significantly older and with more comorbidities in all three waves (Table 5 and Tables S3–S5). Considering the confounding by age, gender, and comorbidity, patients in the second and third waves showed a reduction in severe outcomes (ICU hospitalization or death) compared with patients in the first wave (OR: 0.75, 95% CI: 0.71–0.79; OR: 0.67, 95% CI: 0.63–0.72, respectively) (Figure 4). 

## 4. Discussion

Many authors described the pandemic trends in editorials or opinion papers [18,22,23], and registries have collected data from different COVID-19 waves in 2020 [12,13,24,25], but this is the first observational study that collected clinical data on a region-wide cohort in Italy, evaluating about 245,000 SARS-CoV-2 infected subjects in the first three waves since the beginning of the pandemic. While in Italy, the first wave overwhelmed an unprepared health care system until strict restrictions were imposed at the national level [2], organizational improvement was achieved over time, and policies were targeted at the regional level throughout the following waves [26]. Moreover, as depicted in Figure 1, in the third wave, vaccination started to be implemented, and new therapies (e.g.,: monoclonal antibodies and antiviral drugs) were used.

Our study’s first evidence is the higher number of cases in the second wave, which outnumbered by a five-factor the first one and was double that of the third one [8,9,10,26,27]. The first wave in Italy was affected by an underestimation of cases, which could have been six times lower than the true prevalence, as described by De Natale et al. [28]. The improvement in testing capacity was achieved by increasing the number of laboratories performing RT-PCR and the approval of the use of rapid antigenic testing in January 2021. Nevertheless, the strict lockdown strategy during the first wave could have played a role in reducing the prevalence and shortening the “peak period”, as demonstrated by other authors [18,27], while in the second one, restrictive measures were milder. 

Patients with COVID-19 were significantly younger in the second and third waves, reflecting a diffusion of the illness in the active working population, whereas cases in nursing-home residents prevailed at the end of the first wave. After several COVID-19 outbreaks in nursing homes, in April 2020, access for visitors was forbidden, and isolation and tracking protocols for staff and patients were implemented to reduce SARS-CoV-2 transmission. 

The decrease in the median age of affected patients over time was described in France, Spain, and the USA [11,15,17] in contrast to Germany [29]. Nevertheless, over time we observed a similar need for hospitalization in people aged over 70 and a higher rates of comorbid conditions in admitted patients compared with outpatients, both associated with a poor prognosis [11,12,13,15,17,26]. 

Despite the absolute number of cases (RT-PCR positive patients) and the massive reorganization of the Piedmont hospitals [4,8,26], in the second and third waves, we observed only a slight increase in hospital admissions that, due to the dramatic increase in asymptomatic patients, resulted in a reduction in the percentage of admitted patients. A higher awareness of Emergency Department Physicians in diagnosing and staging COVID-19 disease and a better organization for out-of-hospital COVID care (with the creation of special units to treat COVID patients at home) are possible explanations [26].

We observed that the admission of patients with a Charlson index of more than 2 reduced over time because of the related observed reduction in comorbid burden in the whole cohort [11,15,17]. Nevertheless, in the third wave, we observed a reduced hospitalization of patients with many comorbid conditions in accordance with recent evidence that older patients could be treated safely at home or in nursing homes [30] but also for the possible effect of prioritizing vaccination to frail patients. Moreover, this latter effect is likely the cause of the strong reduction in SARS-CoV-2 infections observed in subjects more than 85 years old and in patients with dementia. 

Similar to other authors [11,12,15], in the second and third waves, we observed a slight reduction in the percentage of patients hospitalized in ICUs in comparison with the first wave, with a parallel reduction in invasive mechanical ventilation and tracheostomy. 

On the contrary, the group of patients treated with CPAP and non-invasive ventilation increased over time, both in ICUs and in high dependency units and low-intensity-of-care wards, in line with other observations [11,15]. This trend toward the increased use of CPAP out of the ICU described the massive implementation in resource availability, staff education, and better knowledge about ventilation and its weaning [31]. During the third wave, we observed a trend toward the greater use of CPAP in and out of the ICU setting and a moderate increase in intubation and invasive ventilation. Little data exist about the third wave, as many studies end in the year 2020 [11,12,13], in January 2021 [15,17,29], and others consider the second and third waves as a continuum [11,12,13,15,29]. The differences we observed in the third wave suggest that patients with COVID-19 diagnosed in the spring of 2021 were younger, severely ill, and treated for a more severe respiratory failure, possibly because they were infected by the delta variant or because of age-related differences in the immune response. 

The length of hospital stay and in-hospital mortality, both in the ICU and in other hospital wards, reduced over time in our cohort as well as being observed elsewhere [11,12,15,17,29]. We hypothesize that this reduction is related to the effect of therapeutic improvements in-between these epidemic waves [11,15,27,31] and the effect of resource optimization due to a better understanding of COVID-19 illness. Organizational improvements (the availability of a higher number of hospital high dependency unit beds and the availability of low-intensity beds in external facilities) reduced the overload of ICUs and hospitals, patients were admitted at the right time during COVID-19’s natural history, and in the right place, both clinical decisions and resource allocation were more efficient. The reduction in overall mortality in outpatients and inpatients reflects this process, which was also described in other countries [11,12,15]. Interestingly, we also observed a reduction in mortality also in more severe cases, such as those admitted to ICUs, whereas in other studies, ICU mortality was persistently higher [11] or not separately described [12,15,17]. 

Examining the subgroup of patients with a poor outcome (death), we observed an increase in mortality that occurs every ten years, with the median age for death always around 80 and a threefold higher risk of death for those over 85. In the third wave, patients who died tended to be younger and concurrently have fewer comorbidities. 

Male gender and the presence of comorbid conditions are associated with death; nevertheless, patients infected in the second and third waves showed an independent reduction in the risk of death. Moreover, in the spring of 2021, the proportion of deceased patients with cardiovascular, renal, cerebrovascular diseases, and dementia significantly decreased, both in the inpatient group and in the overall population. This pattern could be the effect of the Italian government vaccination policy that chose to prioritize vaccination of elderly and frail patients and health care workers. 

Our study has the strength of collecting a broad sample including all of the COVID-19 positive patients of the Piedmont region, allowing some considerations on the effect of organizational improvements that were decided at the regional level. 

Moreover, we tried to observe trends in different subgroups (admitted patients vs. outpatients, admitted in ICU vs. regular wards) to understand differences in resource allocation and mortality in different intensities of care settings and patients with different illness severity. 

Our study has limitations. Firstly, the first wave was affected by an underestimation of positive cases due to the novelty of the emergency, an overwhelmed health care system, and the challenge of tracking. The diagnosis happened more often in patients with severe disease, whereas mild cases with reduced symptoms were underdiagnosed. The analysis of the subgroup of hospitalized patients solved this bias. 

Another limitation is the retrospective nature of this study, which resulted in a possible under-recording of therapeutic procedures on the chart collection system, especially in times of emergency. Nevertheless, this bias is probably equally distributed in the entire sample; we searched for many different codes to avoid missing or misclassifying a procedure. We chose to restrict our analysis to the strongest outcomes and to the procedures with the less possible misinterpretations, taking into account how our health care system of hospital retribution per patient (the ICD-9/DRG system) works [32].

## 5. Conclusions

Our study, the first that evaluated using a broad sample, the clinical characteristics of patients affected by COVID-19, confirmed a trend toward younger and healthier patients over time but also showed an independent effect of the period on mortality and ICU admission. The natural course of the pandemic, the appearance of new viral variants, the starting of vaccination, and the improvements in the tracking of new cases and patient management have influenced these trends. Our analysis of the interventions performed on inpatients allowed us to suggest a role also for organizational improvements (resource availability and more effective patient care) in reducing poor outcomes.

## Figures and Tables

**Table 1:** Demographic characteristics, comorbid conditions, hospitalization, intensive care unit (ICU) admission, and death from COVID-19 of subjects tested positive for SARS-CoV-2, stratifying by waves.

	First Wave (1 March–15 April 2020) N/Day ^a^ (%)	Second Wave (15 October–15 December 2020) N/Day ^a^ (%)	Third Wave (1 March–15 April 2021) N/Day ^a^ (%)	*p*-Value ^b^
**Age group**				
**≤65**	209.0 (51.6%)	1797.3 (75.9%)	1397.0 (78.5%)	<0.001
**66–75**	55.3 13.7%)	216.4 (9.2%)	196.4 (11.1%)	
**76–85**	76.6 (18.9%)	204.1 (8.6%)	133.2 (7.5%)	
**86+**	63.8 (15.8%)	148.5 (6.3%)	52.2 (2.9%)	
**Gender**				
**F**	221.6 (54.7%)	1275.8 (53.9%)	900.3 (50.6%)	<0.001
**M**	183.2 (45.3%)	1090.6 (46.1%)	878.5 (49.4%)	
**Charlson Comorbidity Index**				
**0**	238.7 (59.0%)	1725.8 (72.9%)	1343.5 (75.5%)	<0.001
**1**	89.5 (22.1%)	436.1 (18.4%)	317.3 (17.8%)	
**2–3**	57.8 (14.3%)	165.3 (7.0%)	98.8 (5.6%)	
**4+**	18.7 (4.6%)	39.2 (1.7%)	19.2 (1.1%)	
**Chronic Obstructive Pulmonary Disease (COPD)**				
**No**	328.4 (81.1%)	2019.2 (85.3%)	1529.7 (86.0%)	<0.001
**Yes**	76.4 (18.9%)	347.1 (14.7%)	249.1 (14.0%)	
**Cardiovascular disease**				
**No**	327.9 (81.0%)	2151.7 (90.9%)	1655.8 (93.1%)	<0.001
**Yes**	76.9 (19.0%)	214.7 (9.1%)	123.0 (6.9%)	
**Heart failure**				
**No**	393.9 (97.3%)	2344.1 (99.1%)	1768.9 (99.5%)	<0.001
**Yes**	10.9 (2.7%)	22.3 (0.9%)	9.8 (0.5%)	
**Coronary artery disease**				
**No**	385.5 (95.2%)	2314.8 (97.8%)	1747.1 (98.2%)	<0.001
**Yes**	19.3 (4.8%)	51.6 (2.2%)	31.6 (1.8%)	
**Cardiomyopathy**				
**No**	385.9 (95.3%)	2328.7 (98.4%)	1761.8 (99.0%)	<0.001
**Yes**	18.9 (4.7%)	37.7 (1.6%)	17.0 (1.0%)	
**Diabetes**				
**No**	340.4 (84.1%)	2161.8 (91.3%)	1647.6 (92.6%)	<0.001
**Yes**	64.4 (15.9%)	294.6 (8.7%)	131.1 (7.4%)	
**Kidney disease**				
**No**	388.5 (96.0%)	2330.7 (98.5%)	1761.0 (99.0%)	<0.001
**Yes**	16.3 (4.0%)	35.7 (1.5%)	17.8 (1.0%)	
**Cerebrovascular disease**				
**No**	379.8 (93.8%)	2311.3 (97.7%)	1755.5 (98.7%)	<0.001
**Yes**	25.0 (6.2%)	55.1 (2.3%)	23.3 (1.3%)	
**Dementia**				
**No**	378.3 (96.2%)	2340.1 (98.9%)	1773.1 (99.7%)	<0.001
**Yes**	15.5 (3.8%)	26.3 (1.1%)	5.7 (0.3%)	
**Neoplasia**				
**No**	384.2 (94.9%)	2303.1 (97.3%)	1735.5 (97.6%)	<0.001
**Yes**	20.6 (5.1%)	63.3 (2.7%)	43.3 (2.4%)	
**Haematologic disease**				
**No**	402.3 (99.4%)	2359.7 (99.7%)	1774.8 (99.8%)	<0.001
**Yes**	2.5 (0.6%)	6.7 (0.3%)	3.9 (0.2%)	
**Immunodeficiency**				
**No**	404.6 (99.9%)	2365.7 (99.9%)	1778.3 (99.9%)	0.55
**Yes**	0.2 (0.1%)	0.7 (0.1%)	0.5 (0.1%)	
**Hospitalization**				
**No**	240.1 (59.3%)	2147.8 (90.8%)	1596.8 (89.8%)	<0.001
**Yes**	164.7 (40.7%)	218.6 (9.2%)	182.0 (10.2%)	
**Intensive care unit (ICU) admission**				
**No**	359.9 (88.9%)	2312.7 (97.7%)	1734.7 (97.5%)	<0.001
**Yes**	44.9 (11.1%)	53.7 (2.3%)	44.1 (2.5%)	
**Death**				
**No**	332.3 (82.1%)	2275.1 (96.1%)	1732.1 (97.4%)	<0.001
**Yes**	72.5 (17.9%)	91.3 (3.9%)	46.6 (2.6%)	

^a^ N/day average number of cases per day; ^b^ Comparisons among the three waves tested by Chi-square or Fisher test.

**Table 2:** Demographic characteristics, comorbid conditions, intensive care unit (ICU) admission, and death from COVID-19 of subjects tested positive for SARS-CoV-2, stratifying by hospitalization and waves.

	First Wave (1 March–15 April 2020)		Second Wave (15 October–15 December 2020)		Third Wave (1 March–15 April 2021)		
	No Admission N/Day ^a^ (%)	Admission N/Day ^a^ (%)	No Admission N/Day ^a^ (%)	Admission N/Day ^a^ (%)	No Admission N/Day ^a^ (%)	Admission N/Day ^a^ (%)	*p*-Value ^b^
**Age group**							
**≤65**	150.4 (62.6%)	58.7 (35.6%) ***^,c^	1731.8 (80.6%)	65.6 (30.0%) ***^,d^	1331.7 (83.4%)	65.3 (35.9%) ***^,e^	<0.001
**66–75**	19.5 (8.1%)	35.8 (21.8%)	168.4 (7.9%)	48.0 (22.0%)	148.7 (9.3%)	47.7 (26.2%)	
**76–85**	30.8 (12.9%)	45.8 (27.8%)	137.2 (6.4%)	66.9 (30.6%)	85.4 (5.3%)	47.8 (26.3%)	
**86+**	39.4 (16.4%)	24.4 (14.8%)	110.4 (5.1%)	38.1 (17.4%)	31.0 (2.0%)	21.1 (11.6%)	
**Gender**							
**F**	155.5 (64.8%)	66.0 (40.1%) ***^,c^	1196.5 (55.2%)	89.3 (40.9%) ***^,d^	823.8 (51.6%)	76.5 (42.0%) ***^,e^	0.04
**M**	84.6 (35.2%)	98.7 (59.9%)	961.4 (44.8%)	128.2 (59.1%)	773.0 (48.4%)	105.5 (58.0%)	
**Charlson Comorbidity Index**							
**0**	159.0 (66.2%)	79.6 (48.4%) ***^,c^	1624.0 (75.6%)	101.8 (46.6%) ***^,d^	1247.6 (78.1%)	96.9 (52.7%) ***^,e^	<0.001
**1**	45.4 (18.9%)	44.1 (25.8%)	374.5 (17.4%)	61.6 (28.2%)	268.6 (16.8%)	48.7 (26.7%)	
**2–3**	27.5 (11.5%)	30.3 (18.4%)	123.9 (5.8%)	41.3 (18.9%)	69.4 (4.4%)	29.4 (16.2%)	
**4+**	8.1 (3.4%)	10.6 (6.4%)	25.4 (1.2%)	13.8 (6.3%)	11.2 (0.7%)	8.0 (4.4%)	
**ICU admission**							
**No**		119.8(72.8%) ***^,c^		164.9 (75.5%) ***^,d^		137.9 (75.8%) ***^,e^	<0.001
**Yes**		44.9 (27.2%)		53.7 (24.6%)	..	44.1 (24.2%)	
**Death**							
**No**	216.3 (90.1%)	116.0 (70.5%) ***^,c^	2111.1 (98.3%)	164.0 (75.0%) ***^,d^	1585.1 (99.3%)	147.0 (80.8%) ***^,e^	<0.001
**Yes**	23.8 (9.9%)	48.7 (29.5%)	36.7 (1.7%)	54.6 (25.0%)	11.7 (0.7%)	35.0 (19.2%)	

^a^ N/day average number of cases per day; ^b^ Comparisons of hospital admitted patients among the three waves tested by Chi-square or Fisher test; ^c^ Comparison between hospitalized and not hospitalized subjects during the first wave tested by Chi-square or Fisher test; ^d^ Comparison between hospitalized and not hospitalized subjects during the second wave tested by Chi-square or Fisher test; ^e^ Comparison between hospitalized and not hospitalized subjects during the third wave tested by Chi-square or Fisher test; *** *p* < 0.001.

**Table 3:** Demographic characteristics, comorbid conditions, and death from COVID-19 of patients hospitalized for SARS-CoV-2, stratifying by admission to intensive care unit (ICU) and waves.

	First Wave (1 March–15 April 2020)		Second Wave (15 October–15 December 2020)		Third Wave (1 March–15 April 2021)		
	No ICU N/Day ^a^ (%)	ICU N/Day ^a^ (%)	No ICU N/Day ^a^ (%)	ICU N/Day ^a^ (%)	No ICU N/Day ^a^ (%)	ICU N/Day ^a^ (%)	*p*-Value ^b^
**Age group**							
**≤65**	38.3 (32.8%)	19.4 (43.2%) ***^,c^	47.8 (29.0%)	17.8 (33.2%) ***^,d^	47.2 (34.2%)	18.2 (41.2%) ***^,e^	<0.001
**66–75**	22.5 (18.8%)	13.3 (29.7%)	31.2 (18.9%)	16.8 (31.2%)	32.5 (23.6%)	15.2 (34.5%)	
**76–85**	36.0 (30.0%)	9.8 (21.9%)	51.9 (31.5%)	15.0 (27.9%)	39.0 (28.3%)	8.8 (20.0%)	
**86+**	22.1 (18.4%)	2.3 (5.2%)	33.9 (20.6%)	4.1 (7.7%)	19.2 (13.9%)	1.9 (4.3%)	
**Gender**							
**F**	52.9 (44.1%)	13.2 (29.4%) ***^,c^	72.4 (43.9%)	17.0 (31.6%) ***^,d^	60.7 (44.0%)	15.8 (35.9%) ***^,e^	<0.001
**M**	67.0 (55.9%)	31.7 (70.6%)	92.5 (56.1%)	36.7 (68.4%)	77.2 (56.0%)	28.3 (64.1%)	
**Charlson Comorbidity Index**							
**0**	56.6 (47.2%)	23.1 (51.4%) **^,c^	76.9 (46.6%)	25.0 (46.5%) *^,d^	71.9 (52.1%)	24.0 (54.5%) **^,e^	<0.001
**1**	32.1 (26.8%)	12.0 (26.7%)	45.7 (27.7%)	16.0 (29.8%)	36.5 (26.5%)	12.2 (27.6%)	
**2–3**	23.0 (19.2%)	7.4 (16.4%)	31.6 (19.2%)	9.7 (18.1%)	23.1 (16.8%)	6.3 (14.2%)	
**4+**	8.2 (6.8%)	2.5 (2.5%)	10.7 (6.5%)	3.0 (5.6%)	6.4 (4.6%)	1.6 (3.7%)	
**Death**							
**No**	87.3 (72.8%)	28.7 (64.0%) ***^,c^	128.0 (77.6%)	36.0 (67.0%) ***^,d^	115.2 (83.5%)	31.8 (72.2%) ***^,e^	<0.001
**Yes**	32.5 (27.2%)	16.1 (36.0%)	36.9 (22.4%)	17.7 (33.0%)	22.7 (16.5%)	12.3 (27.8%)	

^a^ N/day average number of cases per day; ^b^ Comparisons of patients admitted to ICU among the three waves tested by Chi-square or Fisher test; ^c^ Comparison between patients admitted in ICU and in other wards (No ICU) during the first wave tested by Chi-square or Fisher test; ^d^ Comparison between patients admitted in ICU and in other wards (No ICU) during the second wave tested by Chi-square or Fisher test; ^e^ Comparison between patients admitted in ICU and in other wards (No ICU) during the third wave tested by Chi-square or Fisher test; *** *p* < 0.001; ** *p* < 0.01; * *p* < 0.05.

**Table 4:** Length of hospital stay and therapeutic interventions performed in patients hospitalized for SARS-CoV-2, in intensive care unit (ICU), and other wards (No ICU), stratifying by waves.

	First Wave (1 March–15 April 2020)			Second Wave (15 October–15 December 2020)			Third Wave (1 March–15 April 2021)			
	Admitted (%)	No ICU N/Day ^a^ (%)	ICU N/Day ^a^ (%)	Admitted (%)	No ICU N/Day ^a^ (%)	ICU N/Day ^a^ (%)	Admitted (%)	No ICU N/Day ^a^ (%)	ICU N/Day ^a^ (%)	*p*-Value ^b^
**Length of hospital stay**										
**Mean (SD)**	21.3 (23.9)	18.8 (19.5)	28.0 (32.0) ***^,c^	18.5 (19.8)	17.0 (17.5)	23.0 (25.1) ***^,d^	18.4 (17.4)	16.4 (15.0)	24.8 (22.4) ***^,e^	<0.001
**Median (IQR)**	14.0 (7.0–28.0)	12.0 (6.0–25.0)	19.0 (9.0–35.0) ***^,c^	13.0 (7.0–22.0)	12.0 (7.0–21.0)	16.0 (9.0–26.0) ***^,d^	14.0 (8.0–23.0)	12.0 (7.0–21.0)	18.0 (11.0–30.0) ***,^e^	<0.001
**Oxygen**										
**No**	57.9 (35.2%)	35.9 (30.0%)	22.0 (49.0%) ***^,c^	78.0 (35.7%)	55.5 (33.7%)	22.5 (41.9%) ***^,d^	67.8 (37.3%)	46.4 (33.7%)	21.4 (48.5%) ***^,e^	<0.001
**Yes**	106.8 (64.8%)	83.9 (70.0%)	22.9 (51.0%)	140.5 (64.3%)	109.3 (66.3%)	31.2 (58.1%)	114.2 (62.7%)	91.5 (66.3%)	22.7 (51.5%)	
**CPAP**										
**No**	128.8 (78.2%)	106.2 (88.6%)	22.6 (50.3%) ***^,c^	168.6 (77.2%)	140.9 (85.5%)	27.7 (51.6%) ***^,d^	122.5 (67.3%)	103.8 (75.3%)	18.7 (42.4%) ***^,e^	<0.001
**Yes**	35.9 (21.8%)	13.7 (11.4%)	22.3 (49.7%)	49.9 (22.8%)	24.0 (14.5%)	26.0 (48.4%)	59.4 (32.7%)	34.1 (24.7%)	25.4 (57.6%)	
**NIV**										
**No**	162.5 (98.7%)	119.3 (99.5%)	43.2 (96.4%) ***^,c^	214.7 (98.2%)	163.3 (99.0%)	51.4 (95.8%) ***^,d^	177.2 (97.4%)	135.6 (98.3%)	41.7 (94.5%) ***^,e^	0.01
**Yes**	2.2 (1.3%)	0.5 (0.5%)	1.6 (3.6%)	3.8 (1.8%)	1.6 (1.0%)	2.2 (4.2%)	4.8 (2.6%)	2.3 (1.7%)	2.4 (5.5%)	
**Intubation**										
**No**	155.9 (94.6%)	..	36.2 (80.7%) ***^,c^	212.8 (97.3%)	..	48.0 (89.5%) ***^,d^	175.3 (96.3%)	..	37.6 (85.4%) ***^,e^	<0.001
**Yes**	8.8 (5.4%)	..	8.7 (19.3%)	5.8 (2.7%)	..	5.6 (10.5%)	6.7 (3.7%)	..	6.4 (14.6%)	
**Invasive ventilation**										
**No**	146.4 (88.9%)	118.8 (99.2%)	27.6 (61.5%) ***^,c^	203.9 (93.3%)	164.3 (99.6%)	39.6 (73.8%) ***^,d^	167.5 (92.0%)	137.3 (99.6%)	30.2 (68.5%) ***^,e^	<0.001
**Yes**	18.3 (11.1%)	1.0 (0.8%)	17.3 (38.5%)	14.7 (6.7%)	0.6 (0.4%)	14.1 (26.2%)	14.5 (8.0%)	0.6 (0.4%)	13.9 (31.5%)	
**Tracheotomy**										
**No**	158.7 (96.3%)	..	38.9 (86.6%) ***^,c^	214.3 (98.0%)	..	49.4 (92.1%) ***^,d^	177.8 (97.7%)	..	40.0 (90.7%) ***^,e^	<0.001
**Yes**	6.0 (3.7%)	..	6.0 (13.4%)	4.3 (2.0%)	..	4.2 (7.9%)	4.2 (2.3%)	..	4.1 (9.3%)	

^a^ N/day average number of patients who underwent an intervention per day; ^b^ Comparisons of patients admitted to ICU among the three waves tested by Chi-square or Fisher test and Kruskal–Wallis or *t*-test; ^c^ Comparison between patients admitted in ICU and in other wards (No ICU) during the first wave tested by Chi-square or Fisher test and Kruskal–Wallis or *t*-test; ^d^ Comparison between patients admitted in ICU and in other wards (No ICU) during the second wave tested by Chi-square or Fisher test and Kruskal–Wallis or *t*-test; ^e^ Comparison between patients admitted in ICU and in other wards (No ICU) during the third wave tested by Chi-square or Fisher test and Kruskal–Wallis or *t*-test; *** *p* < 0.001.

**Table 5:** Demographic characteristics, comorbid conditions, and intensive care unit (ICU) admission of hospitalized for SARS-CoV-2, stratifying by death within 30 days from the first positive swab and waves.

	First Wave (1 March–15 April 2020)		Second Wave (15 October–15 December 2020)		Third Wave (1 March–15 April 2021)		
	No Death N/Day ^a^ (%)	Death N/Day ^a^ (%)	No Death N/Day ^a^ (%)	Death N/Day ^a^ (%)	No Death N/Day ^a^ (%)	Death N/Day ^a^ (%)	*p*-Value ^b^
**Age group**							
**≤65**	54.2 (46.7%)	4.4 (9.1%) ***^,c^	61.3 (37.4%)	4.2 (7.8%) ***^,d^	61.5 (41.9%)	3.8 (10.8%) ***^,e^	<0.001
**66–75**	26.6 (22.9%)	9.3 (19.1%)	39.2 (23.9%)	8.8 (16.2%)	39.6 (26.9%)	8.1 (23.2%)	
**76–85**	25.3 (21.9%)	20.5 (42.0%)	44.2 (26.9%)	22.8 (41.7%)	34.2 (23.3%)	13.6 (38.8%)	
**86+**	9.9 (8.5%)	14.5 (29.8%)	19.3 (11.8%)	18.8 (34.3%)	11.6 (7.9%)	9.5 (27.2%)	
**Gender**							
**F**	47.8 (41.2%)	18.3 (37.6%) **^,c^	68.1 (41.5%)	21.2 (38.8%) **^,d^	62.7 (42.7%)	13.8 (39.3%) *^,e^	0.49
**M**	68.3 (58.8%)	30.4 (62.4%)	95.8 (58.5%)	33.4 (61.2%)	84.3 (57.3%)	21.2 (60.7%)	
**Charlson Comorbidity Index**							
**0**	63.2 (54.5%)	16.4 (33.7%) ***^,c^	82.9 (50.5%)	19.0 (34.7%) ***^,d^	82.1 (55.8%)	13.8 (39.4%) ***^,e^	0.006
**1**	29.6 (25.5%)	14.5 (29.8%)	45.9 (28.0%)	15.7 (28.8%)	38.6 (26.3%)	10.0 (28.7%)	
**2–3**	17.9 (15.4%)	12.4 (25.6%)	27.3 (16.7%)	14.0 (25.7%)	21.4 (14.6%)	8.0 (22.9%)	
**4+**	5.3 (4.6%)	5.3 (10.9%)	7.9 (4.8%)	5.9 (10.8%)	4.9 (3.3%)	3.1 (8.9%)	
**ICU admission**							
**No**	87.3 (75.2%)	32.5 (66.9%) ***^,c^	128.0 (78.0%)	36.9 (67.6%) ***^,d^	115.2 (78.4%)	22.7 (65.0%) ***^,e^	0.18
**Yes**	28.7 (24.8%)	16.1 (33.1%)	36.0 (22.0%)	17.7 (32.4%)	31.8 (21.6%)	12.3 (35.0%)	

^a^ N/day average number of cases per day; ^b^ Comparisons of dead patients among the three waves tested by Chi-square or Fisher test; ^c^ Comparison between alive and dead patients during the first wave tested by Chi-square or Fisher test; ^d^ Comparison between alive and dead patients during the second wave tested by Chi-square or Fisher test; ^e^ Comparison between alive and dead patients during the third wave tested by Chi-square or Fisher test; *** *p* < 0.001; ** *p* < 0.01; * *p* < 0.05.

## Data Availability

Raw data cannot be made freely available because of restrictions imposed by the Ethical Committees which do not allow open/public sharing of data on individuals. However aggregated data are available for other researchers, on request. Requests should be sent to the corresponding author.

## Supplementary Materials

The following supporting information can be downloaded at: https://www.mdpi.com/article/10.3390/jcm11154304/s1, Figure S1: Map showing Italy and Piedmont region; Figure S2: Number of deaths from SARS-CoV-2 registered in Piedmont during the COVID-19 pandemic. (Data source: GitHub-pcm-dpc/COVID-19: COVID-19 Italia-Monitoraggio situazione). Table S1: Comorbid conditions of subjects tested positive for SARS-CoV-2, stratifying by hospital admission and waves; Table S2: Comorbid conditions of patients hospitalized for SARS-CoV-2, stratifying by admission to intensive care unit (ICU) and waves; Table S3: Comorbid conditions of patients hospitalized for SARS-CoV-2, stratifying by death within 30 days from the first positive swab and waves; Table S4: Demographic characteristics, comorbid conditions, hospitalization, and intensive care unit (ICU) admission of subjects tested positive for SARS-CoV-2, stratifying by death within 30 days from the first positive swab and waves; Table S5: Demographic characteristics and comorbid conditions of subjects admitted to the ICU, stratifying by death within 30 days from the first positive swab and waves.
