# Supplementary material for: Improvements throughout the Three Waves of COVID-19 Pandemic: Results from 4 Million Inhabitants of North-West Italy
Source: J Clin Med. 2022 Jul 25;11(15):4304. doi: 10.3390/jcm11154304 (PMC9332615; doi:10.3390/jcm11154304)
Supplement: Supplementary file 1 [file jcm-11-04304-s001.zip › jcm-1804075-supplementary.pdf]

Figure S1: Map showing Italy and Piedmont region

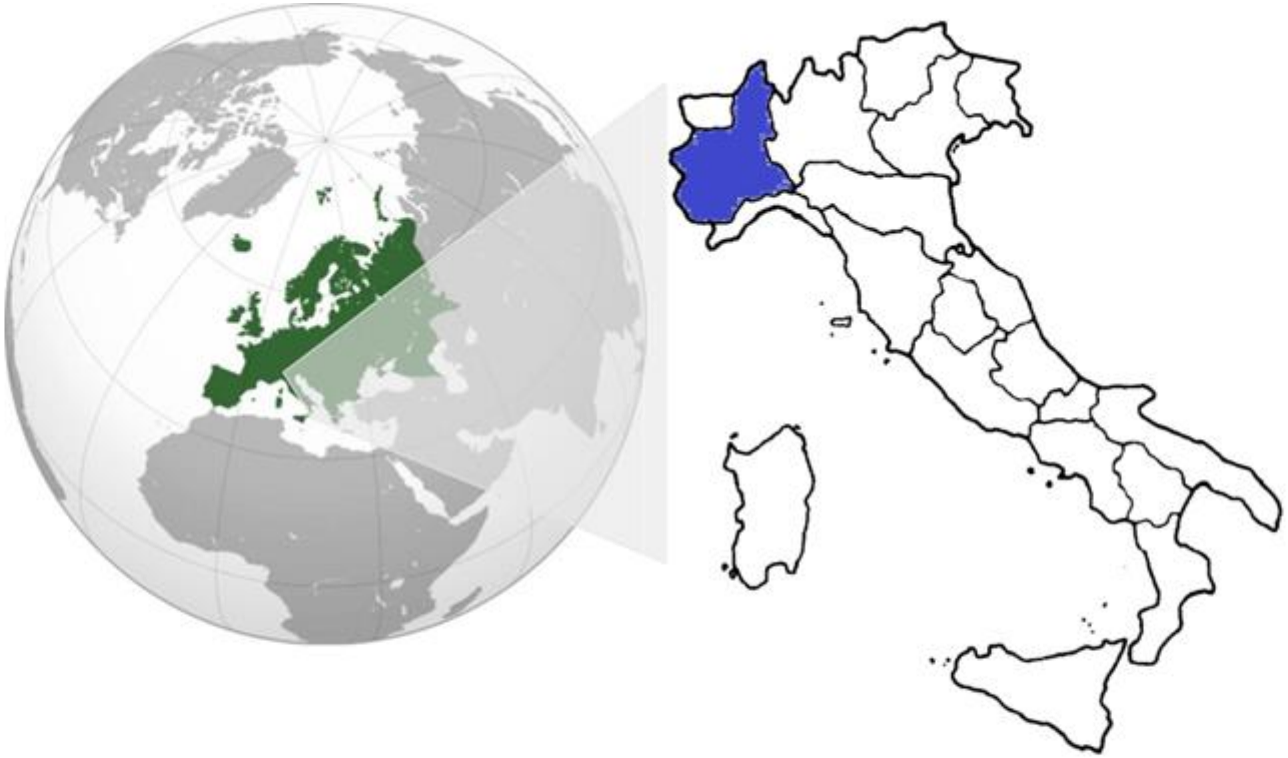

Figure S2: Number of deaths from SARS-CoV-2 registered in Piedmont during the COVID-19 pandemic. (Data source: GitHub - pcm-dpc/COVID-19: COVID-19 Italia - Monitoraggio situazione)

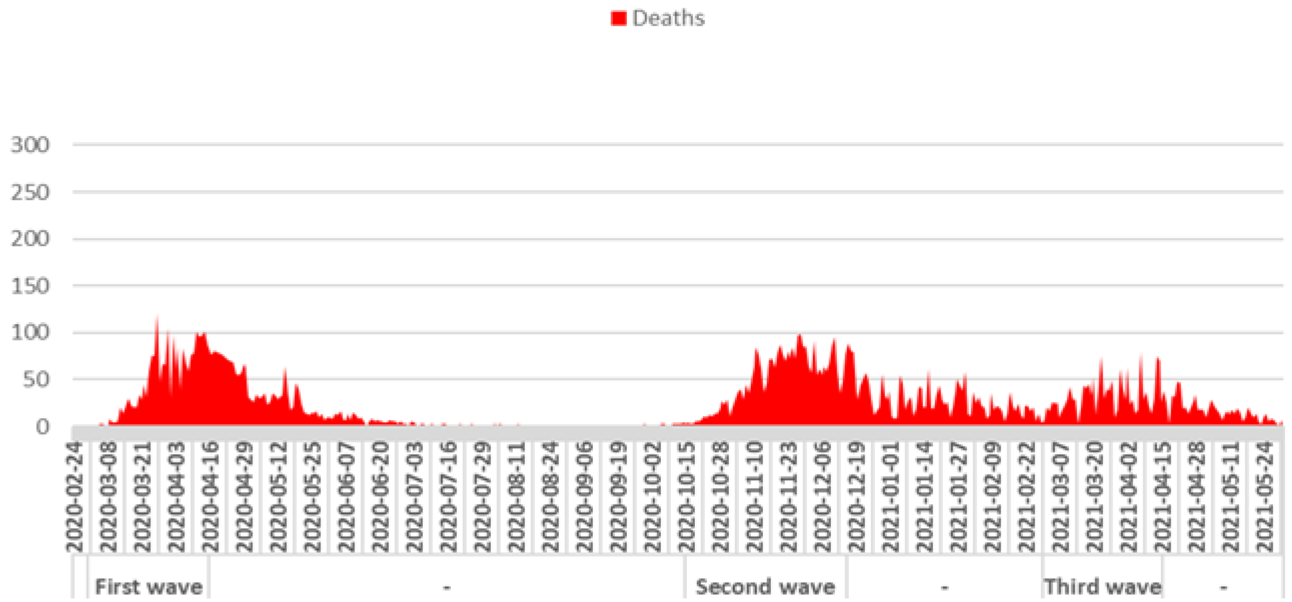

Table S1: Comorbid conditions of subjects tested positive for SARS-CoV-2, stratifying by hospital admission and waves

	**1° Wave** (01/03-15/04/20)		**2° Wave** (15/10-15/12/20)		**3° Wave** (01/03-15/04/21)		
	**No Admission N/days[a] (%)**	**Admission N/days[a] (%)**	**No Admission N/days[a] (%)**	**Admission N/days[a] (%)**	**No Admission N/days[a] (%)**	**Admission N/days[a] (%)**	**P-value[b]**
**Chronic Obstructive Pulmonary Disease (COPD)**							
No	201.3 (83.8%)	127.2 (77.2%)***[c]	1853.7 (86.3%)	165.6 (75.8%)***[d]	1387.4 (86.9%)	142.3 (78.2%)***[e]	<0.001
Yes	38.8 (16.2%)	37.5 (22.8%)	294.2 (13.7%)	53.0 (24.2%)	209.4 (13.1%)	39.7 (21.8%)	
**Cardiovascular disease**							
No	202.6 (84.4%)	125.4 (76.1%)***[c]	1985.7 (92.4%)	165.9 (75.9%)***[d]	1509.6 (94.5%)	146.2 (80.3%)***[e]	<0.001
Yes	37.5 (15.6%)	39.3 (23.9%)	162.1 (7.6%)	52.6 (24.1%)	87.2 (5.5%)	35.8 (19.7%)	
**Heart failure**							
No	234.7 (97.7%)	159.2 (96.6%)***[c]	2133.4 (99.3%)	210.7 (96.4%)***[d]	1591.3 (99.7%)	177.6 (97.6%)***[e]	<0.001
Yes	5.4 (2.3%)	5.5 (3.4%)	14.4 (0.7%)	7.9 (3.6%)	5.5 (0.3%)	4.3 (2.4%)	
**Coronary artery disease**							
No	232.4 (96.8%)	153.1 (92.9%)***[c]	2111.7 (98.3%)	203.0 (92.9%)***[d]	1575.1 (98.6%)	172.0 (94.5%)***[e]	<0.001
Yes	7.7 (3.2%)	11.6 (7.1%)	36.1 (1.7%)	15.5 (7.1%)	21.7 (1.4%)	10.0 (5.5%)	
**Cardiomyopathy**							
No	230.7 (96.1%)	155.1 (94.2%)***[c]	2122.8 (98.8%)	205.9 (94.2%)***[d]	1587.1 (99.4%)	174.7 (96.0%)***[e]	<0.001
Yes	9.4 (3.9%)	9.6 (5.8%)	25.0 (1.2%)	12.7 (5.8%)	9.7 (0.6%)	7.3 (4.0%)	
**Diabetes**							
No	211.9 (88.3%)	128.5 (78.0%)***[c]	1995.0 (92.9%)	166.8 (76.3%)***[d]	1503.0 (94.1%)	144.6 (79.5%)***[e]	<0.001
Yes	28.2 (11.7%)	36.2 (22.0%)	152.8 (7.1%)	51.8 (23.7%)	93.8 (5.9%)	37.3 (20.5%)	
**Kidney disease**							
No	233.2 (97.1%)	155.3 (94.3%)***[c]	2124.4 (98.9%)	206.2 (94.4%)***[d]	1586.8 (99.4%)	173.2 (95.7%)***[e]	<0.001
Yes	6.9 (2.9%)	9.4 (5.7%)	23.4 (1.1%)	12.3 (5.6%)	10.0 (0.6%)	7.8 (4.3%)	
**Cerebrovascular disease**							
No	227.0 (94.6%)	152.8 (92.8%)***[c]	2106.6 (98.1%)	204.7 (93.7%)***[d]	1581.6 (99.0%)	173.9 (95.6%)***[e]	<0.001
Yes	13.1 (5.4%)	11.9 (7.2%)	41.2 (1.9%)	13.8 (6.3%)	15.2 (1.0%)	8.1 (4.4%)	
**Dementia**							
No	230.1 (95.8%)	159.2 (96.6%)**[c]	2127.2 (99.0%)	212.9 (97.4%)***[d]	1593.2 (99.8%)	179.9 (98.8%)***[e]	<0.001
Yes	10.0 (4.2%)	5.5 (3.4%)	20.7 (1.0%)	5.6 (2.6%)	3.6 (0.2%)	2.1 (1.2%)	
**Neoplasia**							
No	231.2 (96.3%)	153.0 (92.9%)***[c]	2100.1 (97.8%)	203.0 (92.9%)***[d]	1565.5 (98.0%)	170.0 (93.4%)***[e]	0.31
Yes	8.9 (3.7%)	11.7 (7.1%)	47.8 (2.2%)	15.5 (7.1%)	31.2 (2.0%)	12.0 (6.6%)	
**Haematologic disease**							
No	239.3 (99.7%)	163.0 (99.0%)***[c]	2143.3 (99.8%)	216.3 (99.0%)***[d]	1594.3 (99.8%)	180.5 (99.2%)***[e]	0.18
Yes	0.8 (0.3%)	1.7 (1.0%)	4.5 (0.2%)	2.2 (1.0%)	2.5 (0.2%)	1.4 (0.8%)	
**Immunodeficiency**							

No	240.1 (99.9%)	164.6 (99.9%)°[c]	2147.3 (99.9%)	218.4 (99.9%)**[d]	1596.4 (99.9%)	181.9 (99.9%)°[e]	0.88
Yes	0.1 (0.1%)	0.1 (0.1%)	0.5 (0.1%)	0.2 (0.1%)	0.4 (0.1%)	0.1 (0.1%)	

[a] N/days average number of cases per day

[b] Comparisons of hospitalized patients among the three waves tested by Chi-square or Fisher test

[c] Comparison between hospitalized and not hospitalized subjects during the first wave tested by Chi-square or Fisher test

[d] Comparison between hospitalized and not hospitalized subjects during the second wave tested by Chi-square or Fisher test

[e] Comparison between hospitalized and not hospitalized subjects during the third wave tested by Chi-square or Fisher test

*** $p < 0.001$; ** $p < 0.01$; * $p < 0.05$; ° $p \geq 0.05$

Table S2: Comorbid conditions of patients hospitalized for SARS-CoV-2, stratifying by admission to intensive care unit (ICU) and waves

	**1° Wave** (01/03-15/04/20)		**2° Wave** (15/10-15/12/20)		**3° Wave** (01/03-15/04/21)		
	**No ICU N/days[a] (%)**	**ICU N/days[a] (%)**	**No ICU N/days[a] (%)**	**ICU N/days[a] (%)**	**No ICU N/days[a] (%)**	**ICU N/days[a] (%)**	**P-value[b]**
**Chronic Obstructive Pulmonary Disease (COPD)**							
No	91.3 (76.2%)	35.9 (80.0%)***[c]	124.4 (75.4%)	41.2 (76.7%°)[d]	108.1 (78.4%)	34.2 (77.6%)[e]	0.02
Yes	28.6 (23.8%)	9.0 (20.0%)	40.5 (24.6%)	12.5 (23.3%)	29.8 (21.6%)	9.8 (22.4%)	
**Cardiovascular disease**							
No	90.0 (75.1%)	35.3 (78.8%)**[c]	123.5 (74.9%)	42.4 (79.1%)***[d]	109.5 (79.4%)	36.7 (83.2%)***[e]	<0.001
Yes	29.8 (24.9%)	9.5 (21.2%)	41.4 (25.1%)	11.2 (20.9%)	28.4 (20.6%)	7.4 (16.8%)	
**Heart failure**							
No	115.4 (96.3%)	43.8 (97.6%)**[c]	158.4 (96.0%)	52.3 (97.5%)***[d]	134.5 (97.5%)	43.2 (98.0%)°[e]	0.51
Yes	4.5 (3.7%)	1.1 (2.4%)	6.5 (4.0%)	1.3 (2.5%)	3.4 (2.5%)	0.9 (2.0%)	
**Coronary artery disease**							
No	111.7 (93.2%)	41.4 (92.2%)°[c]	153.4 (93.0%)	49.7 (92.6%)°[d]	130.2 (94.4%)	41.8 (94.9%)°[e]	0.001
Yes	8.1 (6.8%)	3.5 (7.8%)	11.5 (7.0%)	4.0 (7.4%)	7.7 (5.6%)	2.3 (5.1%)	
**Cardiomyopathy**							
No	112.1 (93.6%)	43.0 (95.8%)***[c]	154.7 (93.8%)	41.2 (95.4%)**[d]	132.2 (95.9%)	42.5 (96.4%)°[e]	0.18
Yes	7.7 (6.4%)	1.9 (4.2%)	10.2 (6.2%)	2.5 (4.6%)	5.7 (4.1%)	1.6 (3.6%)	
**Diabetes**							
No	94.0 (78.5%)	34.4 (76.7%)°[c]	126.5 (76.7%)	40.2 (75.0%)*[d]	109.5 (79.4%)	35.1 (79.7%)°[e]	<0.001
Yes	25.8 (21.5%)	10.5 (23.3%)	38.4 (23.3%)	13.4 (25.0%)	28.4 (20.6%)	9.0 (20.3%)	
**Kidney disease**							
No	112.5 (93.9%)	42.8 (95.4%)**[c]	155.6 (94.4%)	50.6 (94.3%)°[d]	131.7 (95.5%)	42.5 (96.4%)°[e]	0.003
Yes	7.3 (6.1%)	2.0 (4.6%)	9.3 (5.6%)	3.0 (5.7%)	6.2 (4.5%)	1.6 (3.6%)	
**Cerebrovascular disease**							
No	110.1 (91.9%)	42.6 (95.1%)***[c]	153.4 (93.0%)	51.3 (95.6%)***[d]	131.2 (95.2%)	42.6 (96.8%)**[e]	0.02
Yes	9.7 (8.1%)	2.2 (4.9%)	11.5 (7.0%)	2.4 (4.4%)	6.7 (4.8%)	1.4 (3.2%)	
**Dementia**							
No	114.8 (95.8%)	44.3 (98.8%)***[c]	159.6 (96.8%)	53.3 (99.3%)***[d]	136.0 (98.6%)	43.9 (99.6%)**[e]	0.03
Yes	5.0 (4.2%)	0.5 (1.2%)	5.2 (3.2%)	0.4 (0.7%)	1.9 (1.4%)	0.2 (0.4%)	
**Neoplasia**							
No	110.9 (92.6%)	42.1 (93.7%)°[c]	153.4 (93.0%)	49.6 (92.4%)°[d]	127.9 (92.8%)	42.0 (95.4%)***[e]	<0.001
Yes	8.9 (7.4%)	2.8 (6.3%)	11.5 (7.0%)	4.1 (7.6%)	10.0 (7.2%)	2.0 (4.6%)	
**Haematologic disease**							
No	118.7 (99.1%)	44.3 (98.7%)°[c]	163.4 (99.1%)	52.9 (98.6%)*[d]	136.6 (99.1%)	43.9 (99.6%)*[e]	0.001
Yes	1.1 (0.9%)	0.6 (1.3%)	1.5 (0.9%)	0.8 (1.4%)	1.3 (0.9%)	0.2 (0.4%)	

**Immunodeficiency**							
No	119.7 (99.9%)	44.8 (99.9%)°[c]	164.7 (99.9%)	53.6 (99.9%)°[d]	137.8 (99.9%)	44.0 (99.9%)°[e]	0.86
Yes	0.1 (0.1%)	0.1 (0.1%)	0.1 (0.1%)	0.1 (0.1%)	0.1 (0.1%)	0.1 (0.1%)	

[a] N/days average number of cases per day

[b] Comparisons of patients admitted to ICU among the three waves tested by Chi-square or Fisher test

[c] Comparison between patients admitted in ICU and in other wards (No ICU) during the first wave tested by Chi-square or Fisher test

[d] Comparison between patients admitted in ICU and in other wards (No ICU) during the second wave tested by Chi-square or Fisher test

[e] Comparison between patients admitted in ICU and in other wards (No ICU) during the third wave tested by Chi-square or Fisher test

*** $p < 0.001$; ** $p < 0.01$; * $p < 0.05$; ° $p \geq 0.05$

Table S3: Comorbid conditions of patients hospitalized for SARS-CoV-2, stratifying by death within 30 days from the first positive swab and waves

	1° Wave (01/03-15/04/20)		2° Wave (15/10-15/12/20)		3° Wave (01/03-15/04/21)		
	No Death N/days[a] (%)	Death N/days[a] (%)	No Death N/days[a] (%)	Death N/days[a] (%)	No Death N/days[a] (%)	Death N/days[a] (%)	P-value[b]
**Chronic Obstructive Pulmonary Disease (COPD)**							
No	92.3 (79.5%)	34.9 (71.6%)***[c]	127.0 (77.5%)	38.5 (70.6%)***[d]	117.2 (79.7%)	25.1 (71.7%)***[e]	0.62
Yes	23.7 (20.5%)	13.8 (28.4%)	36.9 (22.5%)	16.0 (29.4%)	29.8 (20.3%)	9.9 (28.3%)	
**Cardiovascular disease**							
No	93.3 (80.4%)	32.0 (65.8%)***[c]	129.9 (79.2%)	36.0 (66.0%)***[d]	121.6 (82.7%)	24.6 (70.3%)***[e]	0.005
Yes	22.7 (19.6%)	16.6 (34-2%)	34.0 (20.8%)	18.6 (34.0%)	25.3 (17.3%)	10.4 (29.7%)	
**Heart failure**							
No	113.4 (97.8%)	45.7 (93.9%)***[c]	159.4 (97.2%)	51.3 (94.0%)***[d]	144.5 (98.3%)	33.2 (94.7%)***[e]	0.55
Yes	2.6 (2.2%)	3.0 (6.1%)	4.6 (2.8%)	3.2 (6.0%)	2.5 (1.7%)	1.8 (5.3%)	
**Coronary artery disease**							
No	109.5 (94.4%)	43.6 (89.6%)***[c]	153.9 (93.8%)	49.2 (90.1%)***[d]	140.1 (95.3%)	31.9 (91.1%)***[e]	0.29
Yes	6.5 (5.6%)	5.1 (10.4%)	10.1 (6.2%)	5.4 (9.9%)	6.9 (4.7%)	3.1 (8.9%)	
**Cardiomyopathy**							
No	111.6 (96.2%)	43.5 (89.5%)***[c]	156.9 (95.7%)	49.0 (89.8%)***[d]	142.7 (97.1%)	32.0 (91.3%)***[e]	0.14
Yes	4.4 (3.8%)	5.1 (10.5%)	7.1 (4.3%)	5.6 (10.2%)	4.3 (2.9%)	3.0 (8.7%)	
**Diabetes**							
No	94.1 (81.1%)	34.4 (70.6%)***[c]	128.0 (78.1%)	38.7 (70.9%)***[d]	119.3 (81.1%)	25.4 (72.5%)***[e]	0.40
Yes	21.9 (18.9%)	14.3 (29.4%)	35.9 (21.9%)	15.9 (29.1%)	27.7 (18.9%)	9.6 (27.5%)	
**Kidney disease**							
No	111.1 (95.8%)	44.2 (90.8%)***[c]	157.2 (95.9%)	49.0 (89.8%)***[d]	142.3 (96.8%)	31.9 (91.2%)***[e]	0.21
Yes	4.9 (4.2%)	4.5 (9.2%)	6.7 (4.1%)	5.6 (10.2%)	4.7 (3.2%)	3.1 (8.8%)	
**Cerebrovascular disease**							
No	109.9 (94.7%)	42.9 (88.2%)***[c]	155.9 (95.1%)	48.8 (89.4%)***[d]	141.4 (96.2%)	32.5 (92.7%)***[e]	<0.001
Yes	6.2 (5.3%)	5.7 (11.8%)	8.1 (4.9%)	5.8 (10.6%)	5.5 (3.8%)	2.5 (7.3%)	
**Dementia**							
No	113.6 (97.9%)	45.6 (93.7%)***[c]	161.4 (98.4%)	51.6 (94.5%)***[d]	145.6 (99.1%)	34.3 (97.9%)***[e]	<0.001
Yes	2.5 (2.1%)	3.1 (6.3%)	2.6 (1.6%)	3.0 (5.5%)	1.4 (0.9%)	0.7 (2.1%)	
**Neoplasia**							
No	109.2 (94.1%)	43.8 (90.0%)***[c]	153.6 (93.7%)	49.4 (90.5%)***[d]	138.0 (93.9%)	31.9 (91.2%)***[e]	0.49
Yes	6.8 (5.9%)	4.8 (10.0%)	10.3 (6.3%)	5.2 (9.5%)	8.9 (6.1%)	3.1 (8.8%)	
**Haematologic disease**							
No	115.1 (99.2%)	47.9 (98.5%)**[c]	162.5 (99.1%)	53.9 (98.7%)°[d]	146.0 (99.3%)	34.5 (98.7%)**[e]	0.75
Yes	1.0 (0.8%)	0.7 (1.5%)	1.5 (0.9%)	0.7 (1.3%)	1.0 (0.7%)	0.5 (1.3%)	
**Immunodeficiency**							
No	115.9 (99.9%)	48.7 (99.9%)°[c]	163.9 (99.9%)	54.5 (99.8%)°[d]	146.9 (99.9%)	35.0 (100%)°[e]	0.28

Yes	0.1 (0.1%)	0.1 (0.1%)	0.1 (0.1%)	0.1 (0.2%)	0.1 (0.1%)	0 (0%)	

[a] N/days average number of cases per day
[b] Comparisons of dead patients among the three waves tested by Chi-square or Fisher test
[c] Comparison between alive and dead patients during the first wave tested by Chi-square or Fisher test
[d] Comparison between alive and dead patients during the second wave tested by Chi-square or Fisher test
[e] Comparison between alive and dead patients during the third wave tested by Chi-square or Fisher test
*** $p < 0.001$; ** $p < 0.01$; * $p < 0.05$; ° $p \geq 0.05$

Table S4: Demographic characteristics, comorbid conditions, hospitalization, and intensive care unit (ICU) admission of subjects tested positive for SARS-CoV-2, stratifying by death within 30 days from the first positive swab and waves

	1° Wave (01/03-15/04/20)		2° Wave (15/10-15/12/20)		3° Wave (01/03-15/04/21)		
	No Death N/days[a] (%)	Death N/days[a] (%)	No Death N/days[a] (%)	Death N/days[a] (%)	No Death N/days[a] (%)	Death N/days[a] (%)	P-value[b]
**Age group**							
≤ 65	203.1 (61.1%)	5.9 (8.2%)***[c]	1791.7 (78.7%)	5.6 (6.2%)***[d]	1392.2 (80.4%)	4.7 (10.2%)***[e]	<0.001
66-75	42.9 (12.9%)	12.4 (17.1%)	204.3 (9.0%)	12.2 (13.3%)	186.6 (10.8%)	9.8 (21.0%)	
76-85	48.1 (14.5%)	28.5 (39.3%)	169.6 (7.5%)	34.6 (37.8%)	115.5 (6.6%)	17.8 (38.1%)	
86+	38.1 (11.5%)	25.6 (35.4%)	109.5 (4.8%)	39.0 (42.7%)	37.8 (2.2%)	14.3 (30.7%)	
**Gender**							
F	190.7 (57.4%)	39.8 (42.6%)***[c]	1232.7 (54.2%)	43.1 (47.2%)***[d]	881.6 (50.9%)	18.7 (40.0%)***[e]	<0.001
M	141.6 (42.6%)	41.6 (57.4%)	1042.4 (45.8)	48.2 (52.8%)	850.5 (49.1%)	28.0 (60.0%)	
**Charlson Comorbidity Index**							
0	213.8 (64.3%)	24.9 (34.3%)***[c]	1693.3 (74.4%)	32.5 (35.6%)***[d]	1324.8 (76.5%)	18.7 (40.0%)***[e]	<0.001
1	69.4 (20.9%)	20.2 (27.8%)	410.1 (18.0%)	26.0 (28.5%)	303.9 (17.5%)	13.4 (28.7%)	
2-3	38.6 (11.6%)	19.3 (26.6%)	142.2 (6.3%)	23.9 (25.2%)	88.3 (5.1%)	10.5 (22.5%)	
4+	10.5 (3.2%)	8.2 (11.3%)	29.5 (1.3%)	9.7 (10.7%)	15.1 (0.9%)	4.1 (8.8%)	
**Chronic Obstructive Pulmonary Disease (COPD)**							
No	276.6 (83.2%)	51.8 (71.5%)***[c]	1953.8 (85.9%)	65.4 (71.6%)***[d]	1496.2 (86.4%)	33.5 (71.7%)***[e]	0.99
Yes	55.7 (16.8%)	20.7 (28.5%)	321.2 (14.1%)	25.9 (28.4%)	235.9 (13.6%)	13.2 (28.3%)	
**Cardiovascular disease**							
No	280.9 (84.5%)	47.0 (64.9%)***[c]	2092.1 (92.0%)	59.5 (65.2%)***[d]	1623.1 (93.7%)	32.7 (70.1%)***[e]	<0.001
Yes	51.4 (15.5%)	25.5 (35.1%)	182.9 (8.0%)	31.8 (34.8%)	109.0 (6.3%)	13.9 (29.9%)	
**Heart failure**							
No	326.2 (98.2%)	67.7 (93.3%)***[c]	2258.5 (99.3%)	85.7 (93.8%)***[d]	1724.7 (99.6%)	44.2 (94.8%)***[e]	0.079
Yes	6.1 (1.8%)	4.8 (6.7%)	16.6 (0.7%)	5.7 (6.2%)	7.4 (0.4%)	2.4 (5.2%)	
**Coronary artery disease**							
No	320.2 (96.4%)	65.3 (90.0%)***[c]	2231.7 (98.1%)	83.1 (91.0%)***[d]	1704.5 (98.4%)	42.6 (91.4%)***[e]	0.19
Yes	12.1 (3.6%)	7.2 (10.0%)	43.4 (1.9%)	8.2 (9.0%)	27.6 (1.6%)	4.0 (8.6%)	
**Cardiomyopathy**							
No	321.5 (96.7%)	64.4 (88.8%)***[c]	2247.0 (98.8%)	81.7 (89.5%)***[d]	1719.1 (99.2%)	42.7 (91.4%)***[e]	0.006
Yes	10.8 (3.3%)	8.1 (11.2%)	28.1 (1.2%)	9.6 (10.5%)	13.0 (0.8%)	4.0 (8.6%)	
**Diabetes**							
No	288.6 (86.8%)	51.8 (71.4%)***[c]	2095.5 (92.1%)	66.3 (72.6%)***[d]	1613.5 (93.1%)	34.1 (73.1%)***[e]	0.34
Yes	43.7 (13.2%)	20.7 (28.6%)	179.6 (7.9%)	25.0 (27.4%)	118.6 (6.9%)	12.6 (26.9%)	
**Kidney disease**							
No	323.1 (97.2%)	65.4 (90.2%)***[c]	2248.0 (98.8%)	82.7 (90.5%)***[d]	1718.0 (99.2%)	43.0 (92.1%)***[e]	0.039
Yes	9.2 (2.8%)	7.1 (9.8%)	27.1 (1.2%)	8.7 (9.5%)	14.1 (0.8%)	3.7 (7.9%)	

**Cerebrovascular disease**							
No	316.5 (95.2%)	63.3 (87.4%)***[c]	2231.2 (98.1%)	80.2 (87.8%)***[d]	1712.4 (98.9%)	43.1 (92.4%)***[e]	<0.001
Yes	15.8 (4.8%)	9.2 (12.6%)	43.9 (1.9%)	11.2 (12.2%)	19.7 (1.1%)	3.6 (7.6%)	
**Dementia**							
No	322.5 (97.0%)	66.8 (92.1%)***[c]	2255.6 (99.1%)	84.5 (92.5%)***[d]	1727.7 (99.7%)	45.4 (97.3%)***[e]	<0.001
Yes	9.8 (3.0%)	5.7 (7.9%)	19.4 (0.9%)	6.9 (7.5%)	4.4 (0.3%)	1.2 (2.7%)	
**Neoplasia**							
No	318.9 (96.0%)	65.3 (90.1%)***[c]	2219.9 (97.6%)	83.2 (91.1%)***[d]	1693.0 (97.7%)	42.5 (91.2%)***[e]	0.22
Yes	13.4 (4.0%)	7.2 (9.9%)	55.2 (2.4%)	8.1 (8.9%)	39.2 (2.3%)	4.1 (8.8%)	
**Haematologic disease**							
No	330.8 (99.5%)	71.5 (98.6%)***[c]	2269.3 (99.7%)	90.4 (99.0%)***[d]	1728.8 (99.8%)	46.1 (98.8%)***[e]	0.37
Yes	1.5 (0.5%)	1.0 (1.4%)	5.8 (0.3%)	0.9 (1.0%)	3.3 (0.2%)	0.6 (1.2%)	
**Immunodeficiency**							
No	332.2 (99.9%)	72.5 (99.9%)°[c]	2274.5 (99.9%)	91.3 (99.9%)**[d]	1731.6 (99.9%)	46.7 (100%)°[e]	0.20
Yes	0.2 (0.1%)	0.1 (0.1%)	0.6 (0.1%)	0.1 (0.1%)	0.5 (0.1%)	0 (0%)	
**Hospitalization**							
No	216.3 (65.1%)	23.8 (32.9%)***[c]	2111.1 (92.8%)	36.7 (40.2%)***[d]	1585.1 (91.5%)	11.7 (25.0%)***[e]	<0.001
Yes	116.0 (34.9%)	48.7 (67.1%)	164.0 (7.2%)	54.6 (59.8%)	147.0 (8.5%)	35.0 (75.0%)	
**Admission to ICU**							
No	303.6 (91.3%)	56.4 (77.7%)***[c]	2239.1 (98.4%)	73.6 (80.6%)***[d]	1700.3 (98.2%)	34.4 (73.7%)***[e]	<0.001
Yes	28.7 (8.7%)	16.1 (22.3%)	36.0 (1.6%)	17.7 (19.4%)	31.8 (1.8%)	12.3 (26.3%)	

[a] N/days average number of cases per day
[b] Comparisons of dead subjects among the three waves tested by Chi-square or Fisher test
[c] Comparison between alive and dead subjects during the first wave tested by Chi-square or Fisher test
[d] Comparison between alive and dead subjects during the second wave tested by Chi-square or Fisher test
[e] Comparison between alive and dead subjects during the third wave tested by Chi-square or Fisher test
*** p < 0.001; ** p < 0.01; * p < 0.05; ° p ≥0.05

Table S5: Demographic characteristics and comorbid conditions of subjects admitted to the intensive care unit (ICU), stratifying by death within 30 days from the first positive swab and waves

	1° Wave (01/03-15/04/20)		2° Wave (15/10-15/12/20)		3° Wave (01/03-15/04/21)		
	**No Death N/days[a] (%)**	**Death N/days[a] (%)**	**No Death N/days[a] (%)**	**Death N/days[a] (%)**	**No Death N/days[a] (%)**	**Death N/days[a] (%)**	**P-value[b]**
**Age group**							
≤ 65	16.2 (56.5%)	3.1 (19.4%)***[c]	14.7 (40.9%)	3.1 (17.3%)***[d]	15.2 (47.6%)	3.0 (24.5%)***[e]	<0.001
66-75	7.8 (27.3%)	5.5 (34.0%)	11.3 (31.5%)	5.4 (30.7%)	9.8 (30.0%)	5.4 (43.8%)	
76-85	3.9 (13.5%)	6.0 (36.9%)	8.0 (22.1%)	7.0 (39.8%)	5.7 (17.8%)	3.2 (25.7%)	
86+	0.8 (2.7%)	1.6 (9.7%)	2.0 (5.5%)	2.2 (12.2%)	1.2 (3.7%)	0.7 (6.0%)	
**Gender**							
F	8.8 (30.7%)	4.3 (27.0%)°[c]	11.4 (31.6%)	5.6 (31.5%)°[d]	11.9 (37.5%)	3.9 (31.6%)*[e]	0.079
M	19.9 (69.3%)	11.8 (73.0%)	24.6 (68.4%)	12.1 (68.5%)	19.9 (62.5%)	8.4 (68.4%)	
**Charlson Comorbidity Index**							
0	16.7 (58.2%)	6.3 (39.4%)***[c]	18.6 (51.7%)	6.4 (36.0%)***[d]	18.7 (58.9%)	5.3 (43.1%)***[e]	0.040
1	7.2 (25.0%)	4.8 (29.8%)	10.3 (28.5%)	5.7 (32.3%)	8.2 (25.7%)	4.0 (32.5%)	
2-3	3.7 (12.7%)	3.7 (23.0%)	5.6 (15.4%)	4.1 (23.5%)	4.1 (12.9%)	2.2 (17.5%)	
4+	1.2 (4.1%)	1.3 (7.8%)	1.6 (4.4%)	1.5 (8.2%)	0.8 (2.5%)	0.8 (6.9%)	
**Chronic Obstructive Pulmonary Disease (COPD)**							
No	23.8 (82.9%)	12.1 (74.8%)***[c]	28.5 (79.2%)	12.7 (71.6%)***[d]	25.4 (80.0%)	8.8 (71.6%)***[e]	0.26
Yes	4.9 (17.1%)	4.1 (25.2%)	7.5 (20.8%)	5.0 (28.4%)	6.4 (20.0%)	3.5 (28.4%)	
**Cardiovascular disease**							
No	24.0 (83.5%)	11.3 (70.3%)***[c]	29.6 (82.2%)	12.9 (72.7%)***[d]	27.0 (84.9%)	9.7 (78.9%)***[e]	0.002
Yes	4.7 (16.5%)	4.8 (29.7%)	6.4 (17.8%)	4.8 (27.3%)	4.8 (15.1%)	2.6 (21.1%)	
**Heart failure**							
No	28.2 (98.1%)	15.6 (96.6%)*[c]	35.3 (98.0%)	17.0 (96.4%)**[d]	31.4 (98.6%)	11.8 (96.3%)**[e]	0.94
Yes	0.5 (1.9%)	0.5 (3.4%)	0.7 (2.0%)	0.6 (3.6%)	0.4 (1.4%)	0.5 (3.7%)	
**Coronary artery disease**							
No	27.0 (94.1%)	14.3 (88.8%)***[c]	33.6 (93.4%)	16.1 (90.8%)**[d]	30.5 (96.0%)	11.3 (92.0%)***[e]	0.13
Yes	1.7 (5.9%)	1.8 (11.2%)	2.4 (6.6%)	1.6 (9.2%)	1.3 (4.0%)	1.0 (8.0%)	
**Cardiomyopathy**							
No	27.9 (97.1%)	15.1 (93.5%)***[c]	34.7 (96.5%)	16.5 (93.1%)***[d]	31.0 (97.3%)	11.5 (94.0%)***[e]	0.81
Yes	0.8 (2.9%)	1.0 (6.5%)	1.3 (3.5%)	1.2 (6.9%)	0.8 (2.7%)	0.7 (6.0%)	
**Diabetes**							
No	23.0 (80.0%)	11.4 (70.7%)***[c]	27.7 (77.0%)	12.5 (70.9%)***[d]	26.1 (82.1%)	9.0 (73.4%)***[e]	0.50

Yes	5.7 (20.0%)	4.7 (29.3%)	8.3 (23.0%)	5.1 (29.1%)	5.7 (17.9%)	3.3 (26.6%)	
**Kidney disease**							
No	27.9 (97.0%)	15.0 (92.7%)***[c]	34.5 (95.9%)	16.1 (91.3%)***[d]	31.0 (97.3%)	11.5 (94.0%)***[e]	0.14
Yes	0.9 (3.0%)	1.2 (7.3%)	1.5 (4.1%)	1.5 (8.7%)	0.8 (2.7%)	0.7 (6.0%)	
**Cerebrovascular disease**							
No	27.5 (95.8%)	15.1 (93.8%)*[c]	34.6 (96.2%)	16.7 (94.2%)*[d]	30.8 (97.0%)	11.8 (96.3%)°[e]	0.12
Yes	1.2 (4.2%)	1.1 (6.2%)	1.4 (3.8%)	1.0 (5.8%)	1.0 (3.0%)	0.5 (3.7%)	
**Dementia**							
No	28.5 (99.2%)	15.8 (98.1%)*[c]	35.8 (99.5%)	17.5 (99.0%)°[d]	31.7 (99.7%)	12.2 (99.1%)°[e]	0.17
Yes	0.2 (0.8%)	0.3 (1.9%)	0.2 (0.5%)	0.2 (1.0%)	0.1 (0.3%)	0.1 (0.9%)	
**Neoplasia**							
No	27.3 (95.0%)	14.8 (91.5%)**[c]	33.7 (93.7%)	15.9 (89.8%)***[d]	30.4 (95.7%)	11.6 (94.7%)°[e]	0.004
Yes	1.4 (5.0%)	1.4 (8.5%)	2.3 (6.3%)	1.8 (10.2%)	1.4 (4.3%)	0.7 (5.3%)	
**Haematologic disease**							
No	28.4 (98.9%)	15.8 (98.2%)°[c]	35.5 (98.7%)	17.4 (98.3%)°[d]	31.7 (99.7%)	12.2 (99.5%)°[e]	0.12
Yes	0.3 (1.1%)	0.3 (1.8%)	0.5 (1.3%)	0.3 (1.7%)	0.1 (0.3%)	0.1 (0.5%)	
**Immunodeficiency**							
No	28.7 (99.8%)	16.1 (100%)°[c]	36.0 (100%)	17.7 (99.8%)°[d]	31.8 (99.9%)	12.3 (100%)°[e]	0.50
Yes	0.1 (0.2%)	0 (0%)	0 (0%)	0.1 (0.2%)	0.1 (0.1%)	0 (0%)	

[a] N/days average number of cases per day

[b] Comparisons of ICU admitted dead patients among the three waves tested by Chi-square or Fisher test

[c] Comparison between ICU admitted alive and dead patients during the first wave tested by Chi-square or Fisher test

[d] Comparison between ICU admitted alive and dead patients during the second wave tested by Chi-square or Fisher test

[e] Comparison between ICU admitted alive and dead patients during the third wave tested by Chi-square or Fisher test

*** $p < 0.001$; ** $p < 0.01$; * $p < 0.05$; ° $p \geq 0.05$
